# Exploring investor-business-market interplay for business success prediction

**DOI:** 10.1186/s40537-023-00723-6

**Published:** 2023-04-16

**Authors:** Divya Gangwani, Xingquan Zhu, Borko Furht

**Affiliations:** Department of Electrical Engineering and Computer Science, Florida Atlantic University, Boca Raton, FL 33431 USA

**Keywords:** Machine learning methods, Investments-business-market, Feature engineering, Success prediction

## Abstract

The success of the business directly contributes towards the growth of the nation. Hence it is important to evaluate and predict whether the business will be successful or not. In this study, we use the company’s dataset which contains information from startups to Fortune 1000 companies to create a machine learning model for predicting business success. The main challenge of business success prediction is twofold: (1) Identifying variables for defining business success; (2) Feature selection and feature engineering based on Investor-Business-Market interrelation to provide a successful outcome of the predictive modeling. Many studies have been carried out using only the available features to predict business success, however, there is still a challenge to identify the most important features in different business angles and their interrelation with business success. Motivated by the above challenge, we propose a new approach by defining a new business target based on the definition of business success used in this study and develop additional features by carrying out statistical analysis on the training data which highlights the importance of investments, business, and market features in forecasting business success instead of using only the available features for modeling. Ensemble machine learning methods as well as existing supervised learning methods were applied to predict business success. The results demonstrated a significant improvement in the overall accuracy and AUC score using ensemble methods. By adding new features related to the Investor-Business-Market entity demonstrated good performance in predicting business success and proved how important it is to identify significant relationships between these features to cover different business angles when predicting business success.

**Graphical Abstract:**

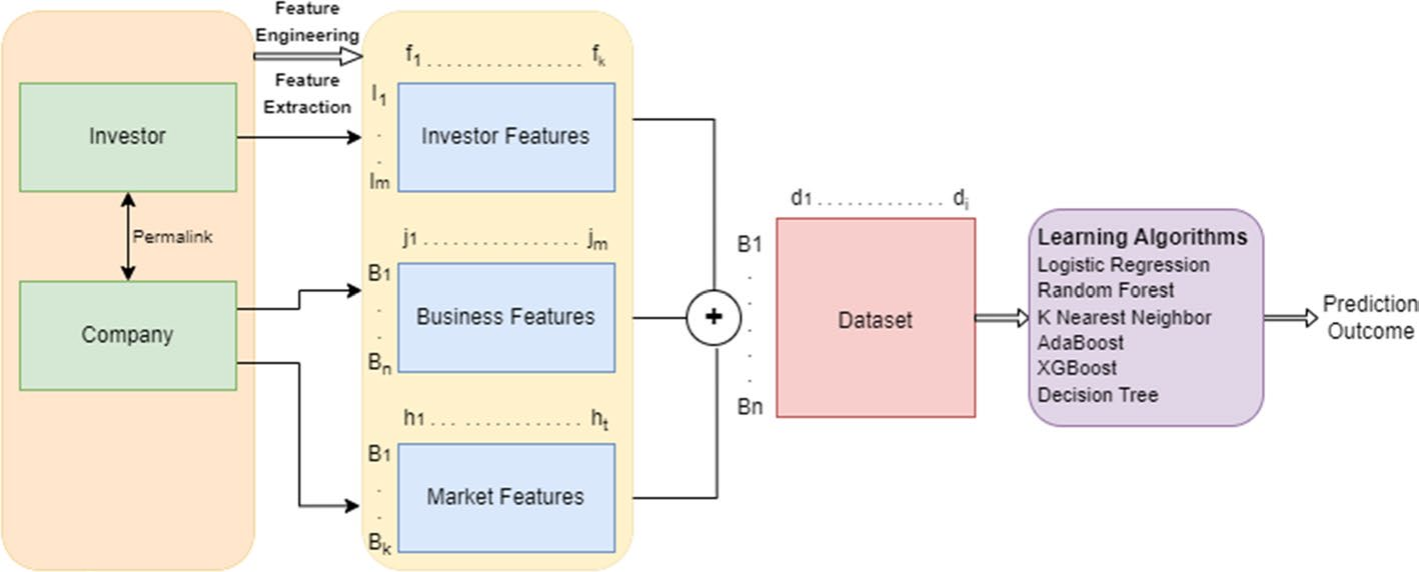

## Introduction

The success of the business is the main reason for the investors, stakeholders and entrepreneurs to stay in the market and grow their business further. This keeps them motivated to come up with new ideas and innovations which is important for the economic growth of the nation. Hence, investors and stakeholders are in constant need of a method that can predict the performance of their business beforehand. It gives them the advantage to invest wisely and compete in the market with an expectation to achieve considerable returns on their investments [1]. Nowadays, many researchers focus on identifying practical tools and methodologies to determine business success factors. There has been a long history of research that tried to analyze the features or factors that make the business successful [2], however, the previous researches needs to be consistent with the literature and features selected in predicting business success. With the ever-changing economy and business dynamics, there is a need to identify the factors that effectively analyze the rise and fall of the business. We aim to bridge the gap in the literature by identifying the most accurate definition of business success such that it brings more clarity in selecting the most critical features from different business angles which are responsible for creating a successful business and developing additional features to demonstrate the importance of selecting suitable features for modeling and predicting business success. In recent years, several small and mid-size companies are gaining attention due to their capability to capture the market and merge with unicorns to achieve more publicity [3]. With millions of investments made by the investors and its rapid increase in achieving unicorn status, it has become even more challenging to predict whether the business will eventually succeed or fail. There are a lot of factors that can affect the performance of the business such as the sector in which the business operates, the number of employees working for the company, skills of the employee, location, size of the company, competition level, and so on. It is difficult to measure all factors and even more challenging to identify several factors which influence the company’s performance.

Recent studies have many limitations due to the use of only specific features to predict business success. For example, [4] utilized only financial features to determine the rise and fall of the business. This would be a trivial solution when other factors are not considered during the evaluation of companies’ performance. Another study highlighted the use of social media marketing to promote business success [5]. It has been observed that by utilizing social media features, business gains more attention and can directly promote their brands and products to customers. Combining social media features with deep learning algorithms improves market captivity for the firm, which ultimately results in success. Recently [6], proposed a framework for applying investment and business features in conjunction when evaluating key criteria for measuring business success. Business factors such as R &D employees, patents in the company, managerial employees, and company valuation which is an important indicator of business success were applied. When a company reaches a valuation of $1 billion it achieves the status of unicorn which distinguishes them from other companies. All these factors together contribute to the success of the business. Therefore it is important to find a correlation between these features in order to accurately predict business success.

Predicting business success is intuitively important and offers great significance to investors and stakeholders as they can effectively utilize the information to attain competitive advantage through timely analysis and accurate prediction.

Machine learning methods have been used in the past to build predictive models for business success and provide corresponding results and suggestions [6–8]. Supervised machine learning methods such as Random Forest, SVM, and Gradient boosting are mostly popularly applied for business prediction using news articles and factual features from company datasets which are publicly available on TechCrunch and Crunchbase websites [9]. In addition, many researchers also proposed neural networks in combination with classification methods to achieve high accuracy when dealing with high cardinality datasets [8]. Despite the growing amount of models built for business success prediction, most of them cannot be applied in practice due to the lack of knowledge about the interrelated features which is an essential requirement for success prediction. Moreover, many methods focus on specific features that define business success [5, 10] and do not take into consideration how other features/factors can play an important role in the decisions making and in turn can result in a biased decision. In addition, many studies [7, 11] gathered data from different sources which included company’s who are still in operating status and does not have enough information to determine their path toward success. Including such information may easily cause issues in trusting the applicability of the results.

In order to accurately predict business success and avoid any kind of bias, there should be a clear definition of success and identify major features and their interrelated sub-features which can be applied in practice to predict business success.

In this paper, we propose a new definition of business success using machine learning techniques to create a predictive model. In our definition, we include companies that have achieved initial public offering (IPO) or have undergone merger and acquisition (M &A) and classify them as successful, and companies that have been closed are classified as failed. Additionally, we use feature engineering techniques to create new features based on three main parities: Investment, Business and Market with a focus on stating the fact that these three entities play a major role in identifying critical factors for business success. Experiments and comparison with baseline demonstrated improved accuracy and AUC score using supervised machine learning algorithms. Ensemble methods, such as Random Forest and XGBoost achieved the best results when compared to other supervised learning methods.

## Business success and investor-business-market interplay

Success in general refers to the achievements obtained either by getting some profit or by fulfilling small goals in life. When we consider business success, the overall definition remains the same, however, there are more factors that affect how we measure business success depending on different business angles.

There is a wide variety of research dedicated to analyzing business success [12–14]. Some use financial indicators are the major factor when predicting success, while others use companies’ demographic information, human resource details, or past financial records to measure a company’s performance. In a recent study carried out in the European market, [15] suggests that utilizing business features such as human resource, demographics, job skills, team size, management, etc. were crucial in distinguishing the success and failure of companies. This research was also extended for U.S market which highlighted the fact that business Human Resource features are capable of detecting success and failure in companies worldwide. Another study [9] focused on the financial indicators for predicting the success of the firms. This is due to the fact that early startups do not have much information to evaluate their path toward progress and in such cases, financial indicators are more reliable for detecting success or failure. Evidence suggests that when a company reaches a new height, for example, has a valuation of $1 billion known as the unicorn, such companies consider different factors for evaluating their growth and cannot be compared to the growth of start-ups or small businesses as they reach to a new stage in the life cycle of a business and experience an exponential rise in the success [16–18]. Hence features such as new innovations, patents, raised amounts by investors, funding amount, market sector, and investor demographics come into play when companies achieve the status of a unicorn. Many studies were conducted on market statistics to see how the market plays an important role in analyzing the growth of the business [19]. Small and medium firms focused on operating on either one industry sector or two with an aim of expanding their business and capturing enough customers so that they can establish their business into one sector firmly, whereas large firms [20]have the capability to capture the market in many industry sectors, such that, even if one sector fails they have more resources and funds to support and grow their businesses in other sectors or industries. For example, a case study done on the European market highlighted the fact that market orientation leads to corporate success in firms [21]. The ultimate goal of achieving success was to narrow down the market and focus on major business dimensions including product cost, customer satisfaction, product environment, technology, and innovations in the business such that the company achieves success in product creation. A successful product in turn leads to successful business due to its capability to capture the market and provide customer satisfaction.

Based on these studies, we conclude that there is not just one factor or a particular set of features that define business success. Several firms are at different stages of their growth and have different factors influencing their business decisions and considering major factors together can contribute in evaluating business success.

In this paper, we consider all types of firms such as small and mid-size firms, startups, unicorns and large companies, when defining business success using machine learning models. In order to measure success, the outcome of the business having a status of either an IPO or Merger and Acquisition (M &A) is considered as a variable of business success and the businesses that have the outcome of closed are considered as failure which becomes our target variable for binary classification problem. We also highlight that several factors or features mentioned in the previous researches are interrelated and cannot be considered separately as the dynamics of business keeps changing but the factors which determine success or failure remain certain. Hence with the above observation, we design an approach to divide these features into three main parities: Investor, Business and Market, and demonstrate how these features together contribute towards evaluating business success irrespective of the type and the size of the business.

### Business success

**Fig. 1:**
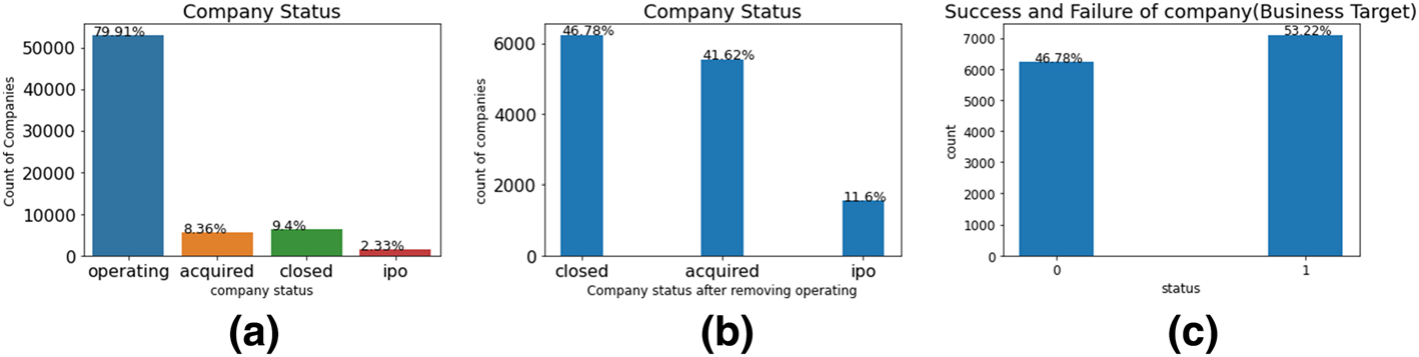
Statistics of company status with respect to four categories (**a**); statistics after removing Operating ones (**b**); **c** statistics of the final labels

The success of the business is defined by the status of the company given in the dataset used for experimentation. The company status is divided into four categories: (1) Operating; (2) IPO; (3) Acquired; and (4) Closed as n in Fig. 1. A company gets the status of operating during its early stages of development or if they are just a survival company and there is not much information available to determine whether these companies will eventually fail or succeed. IPO and Acquired are clear statuses to determine whether these companies have been successful and have received enough funding or have a valuation of a huge amount. When a company goes public they receive the status of IPO which means that they release its portion of funds in the public market with an aim to achieve a huge price gain. Merger and Acquisition (M &A) occurs when a company of the same level gets acquired or merged with another company of a similar level such as Google, Amazon etc. Therefore when a company achieves a status of either IPO or acquired, it is a clear distinction that these companies have enough funding to grow in the market and achieve success whereas the closed status is given to the companies that are no longer operating or have failed to survive. Depending on the company dynamics and having a clear objective of predicting the success of the business, it is important to classify the companies and label them into two categories: success or failure. Based on the dataset used for experiments and keeping the important and relevant feature intact, we selected companies that achieved a status of IPO or Acquisition and labeled them as positive class and the companies that had the status of closed were labeled as negative class 1. The companies that were in the status of operating were excluded from the training set due to the lack of information available to determine whether the companies would be successful or not 1. Keeping such information in the training process includes some kind of bias which may not produce relevant results for comparison. Hence a significant portion of companies were removed from the training set in order to accurately classify and predict business success.

### Investor-business-market triangle

**Fig. 2:**
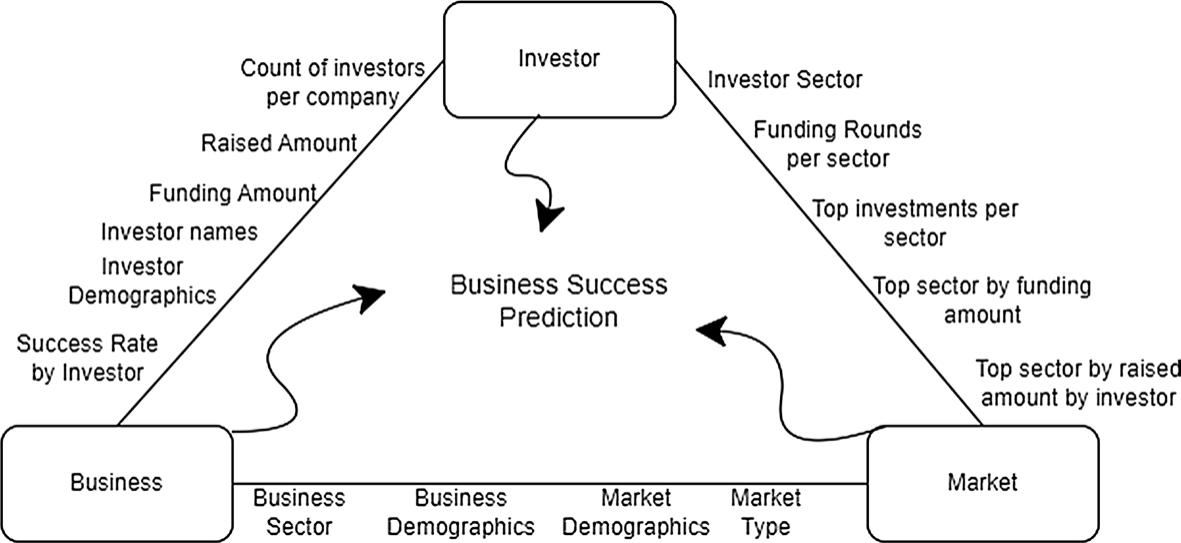
IBM triangle interplay. Investor, Business, and Market are three separate aspects that impact the business success. Texts next to each edge outline representative features we propose to capture the interplay between them

In business prediction, the success of the firms depends on three main entities: Investor, Business, and Market which forms an interrelated triangle as shown in Fig. 2 since these three entities together contribute towards the success of the business. The relationship between these three entities has been supported by wide range of publications [22–25] which shows that when determining whether a business will succeed or fail, these three entities should be taken into account and ignoring any one of of these aspects could lead to an unsatisfactory outcome.

According to the supported literature, investor and the market have a close relationship in contributing towards the economy of the company. In these studies [22, 24], the use of technology in promoting market is directly related to the performance of the business. Information Technology (IT) has changed the ways of how business used to operate. It brings in more employment and investments into the company. Using technology in different market sectors encourages investors to bring more investments into the business which in turn attracts more customers. There are several strategic factors which influence the performance of the company. The four deterministic factors includes: Business demographics, product innovations, market strategy and market trends together analyze the shift in the performance of the company. In the telecommunication industry in Africa [26], statistical analysis was carried out to evaluate the main factors responsible for business performance. In order to evaluate critical factors, customer reviews were taken into account and a business design was prepared to carry out the pros on cons of several factors affecting the industry sector of the company. Focusing on factors such as strategic design, innovation and product creation highlighted the trends in the market and demonstrated how business and market relation led the company to reconsider their failure points and make changes to incorporate different business angles which showed successful innovations in the industry.

A study based on the performance of UK based companies gathered evidence demonstrating how market orientation is directly associated with company’s performance [27]. Factors such as estimated product cost, demographics of company, financial investments etc. contributes towards the performance of the company.

Usually, when a business succeeds it is not because of just one factor, but several factors contribute in combination to the success of the business. As a result, the IBM triangle acts as a shield for the key elements involved in achieving company’s success.

Once we analyze the IBM interplay and define the most important features related to these entities in our dataset, the machine learning model provides enhanced results when predicting business success.

### Investor features

Investor features include three main aspects of the business, Investor demographics, Investor sector and Investor financial information. These three main features helps to answer question such as which business sector has more growth? How many investors invested in the market sector? What is the amount of investments made by the investor? Having answers to such questions provides entrepreneurs with more information about whether the business will get repeatable returns on their investments to better assess their risk of investments into the business. Having investor information related to the business and market sector helps in reducing the risk of uncertainty that comes with every investment made into the business.

The investor demographic features include information of the investor’s location, city, country, and other personal information about the investor. Top investors receive enough recognition over the years such that they always have an edge towards forward-thinking about the growth of the firms. Figure 4 highlights the top 10 investors in the dataset based on the amount of funding raised by the investors. A recent study analyzed how investors’ demographics directly affect the performance of financial sector industry. A stock market industry conducted an annual evaluation to come up with factors affecting their stock price investments [28]. The research showed how demographic factors including age, locality, education level *etc.,* strongly influenced an investor’s decision to buy or sell stocks.

**Fig. 3:**
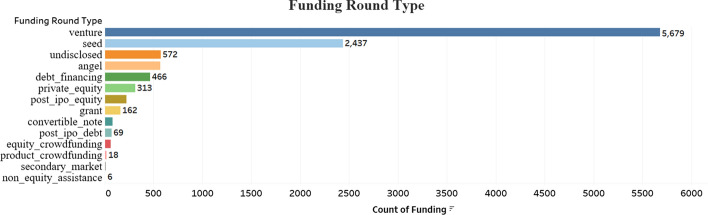
Type of funding rounds provided by investors to the companies

**Fig. 4:**
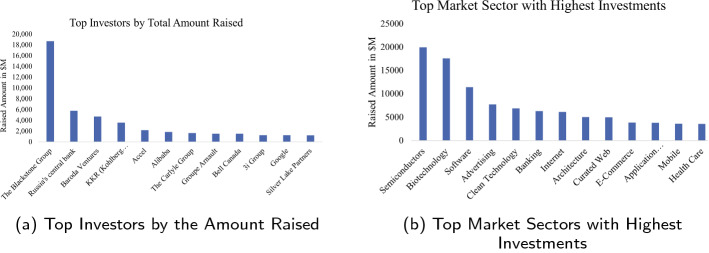
Investor information based on amount raised (**a**), and popular sectors in the industry (**b**)

Investors usually keep a track of recent market trends and customer behavior before making any decision to invest in the company. Hence, evaluating the recent trends in the market sector provides more confidence to the investors to invest into the business. As shown in Fig. 4, we provided statistical analysis on the dataset to include investor features which highlights the top market sectors that received majority of the funding. Semiconductor, Biotechnology and Software are one of the top 3 sectors that received majority of the funding by investors. Investing more money into different markets sector increases the price of those products and this provides an upward momentum for the company to keep growing over the years and maintain their success in the market. Another major aspect of the Investment feature is the financial information about the investor. The financial information includes the capability of an investor to raise more amount in the market and increase the rounds of funding such that the business and the investor achieve maximum profit. Other features such as the funding amount raised by the investor in each sector, type of funding received by the company, returns on investment *etc.,* provides enough evidence to predict the company’s likelihood of success. As shown in Fig. 3, the companies that receives the type of funding such as the Venture Capitalist (VC) funds have higher chances of being public and have faster growth rate as compared to companies backed by either seed or other types of funds [29]. Angel funding is another common type of funds that provides more chances for the company to survive the market risks and growth eventually in terms of more employment, sales and financing [30]. Early startups are in need of such funding and would benefit from a VC fund or any other funding as young firms are more driven by latest technology, ideas and innovations. Investors constantly look for such new innovations and are ready to provide initial funding in exchange for a percentage of profit with these firms [31].

### Business features

Business Features refer to the company’s personal information such as product details, human resources, business demographics, innovations, financial information, and so on. These features are further subdivided into a number of sub-features containing information about the companies. Many studies highlighted the importance of business features when predicting business success. Early-stage startups or small businesses have very little information about major investors or market characteristics due to the limited financial availability to explore larger areas of growth into the business [11]. New ventures or startups rely on the entrepreneur’s techniques and vision to make the business successful. The innovations, new technology, and creativity of an entrepreneur are leading factors for a firm’s success since new innovations or technological changes attract customers and generate profits [32–34]. Another study highlighted that mandating corporate social responsibility on investments made the firm an important aspect in increasing the economy of the nation. A study was done on India’s emerging market which stated that the governing body has a social responsibility to learn and adapt certain policies to create a sustainable environment for the businesses such that firms gain an advantage and achieve profitability over the years [35]. Hence when evaluating the business success of startups or small firms, business features such as business demographics and founders’ vision as well as support from the government plays an important role in giving enough information to make a successful prediction. On the other hand, for large firms, there is a need for more information such as financial features and market trends including business features to evaluate and predict business success. Regardless of the type of firms, business features serves as a common point or a major requirement when predicting business success. A study using business demographic features and human resource features such as company age, team size, number of staff, education level etc., on an established company dataset in US and Europe showed how human resource features were common predictors of success or failure of the firms [15]. This evidence led to the belief that human resource factors needs to be considered as an important resource when predicting business success. Another study on large corporate firms examined several financial indicators for predicting business success [36]. Large firms are more capable of generating profits and hence factors such as returns on investment, capital shares, amount of funding received etc., are key indicators for predicting business success.

**Fig. 5:**
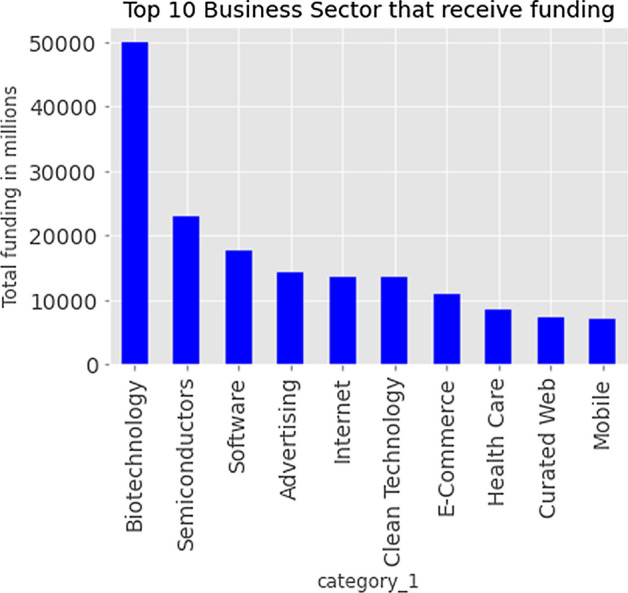
Top 10 business sectors

**Fig. 6:**
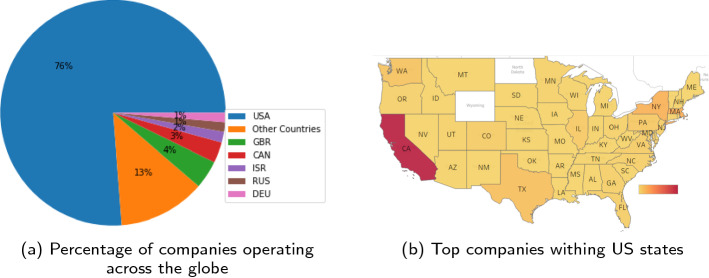
Business Demographic information

Motivated by the previous studies, we identify all the important business features available in the dataset and provide a statistical analysis which gives useful insights of the variables when developing a predictive model. For example, the analysis done on business sectors in the company demonstrates that most of the funding goes to the top business sectors that are in high demand in the market as shown in Fig. 5. Another important factor for growth of the business is the demographics of the company that have been highlighted in Fig. 6, which highlights that U.S has highest number of companies that are either startups or operating and within the U.S, California has the majority of headquarters locations. The sector in which the business operates is one of the important features when predicting the performance of the company as business sectors provide a sustainable environment for the businesses to flourish [37]. With the ever-changing market, it is essential for the companies to keep a track of those changes and shift their investments strategies or switch funds based on the predictive methods used for analyzing market trends.

### Market features

Market features are one of the main aspects of the business that decides the rise and fall of a company. Factors such as market pricing, competitive strength, market size, market digitization, demand, and demographics determine the trends in the market and attract customers to generate market advantage for their business.

Recently, external factors have contributed more towards the shift in the market which has led the entrepreneurs to change their strategies and decisions and share their vision with other stakeholders to keep up with the ups and downs in the business [38]. Market shifts due to external factors such as Covid-19 outbreak, inflation etc, have led to job layoffs, disruption in the commercial sectors, and shut down many high-growing sectors worldwide. For example, the tourism industry have seen a sharp decline during Covid-19 pandemic whereas the pharmaceutical industries have seen a rise in their profit ratios [39].

**Fig. 7:**
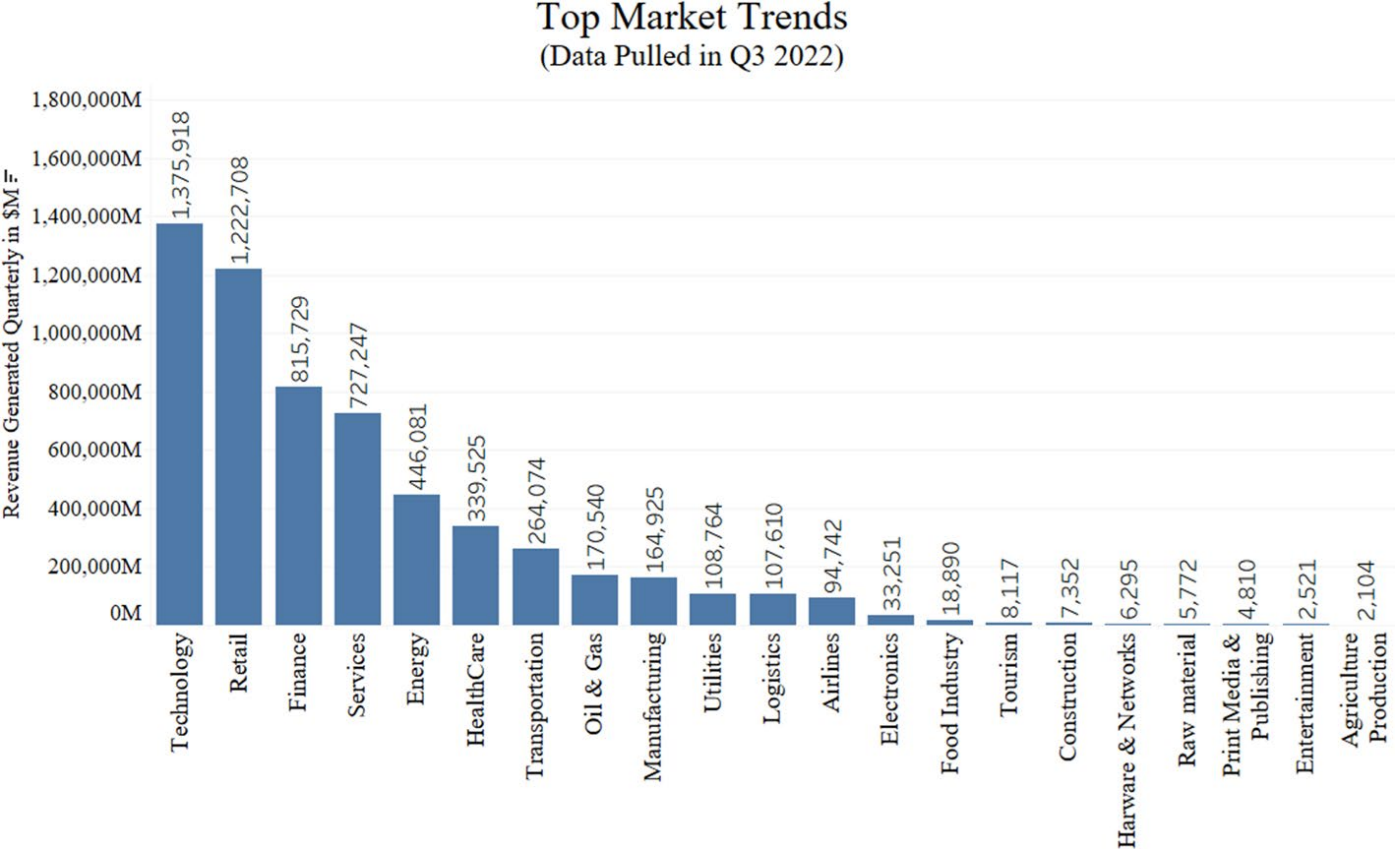
Market trend in 2022 quarter with revenue generated in millions by each sector (the plot only lists popular sectors)

**Fig. 8:**
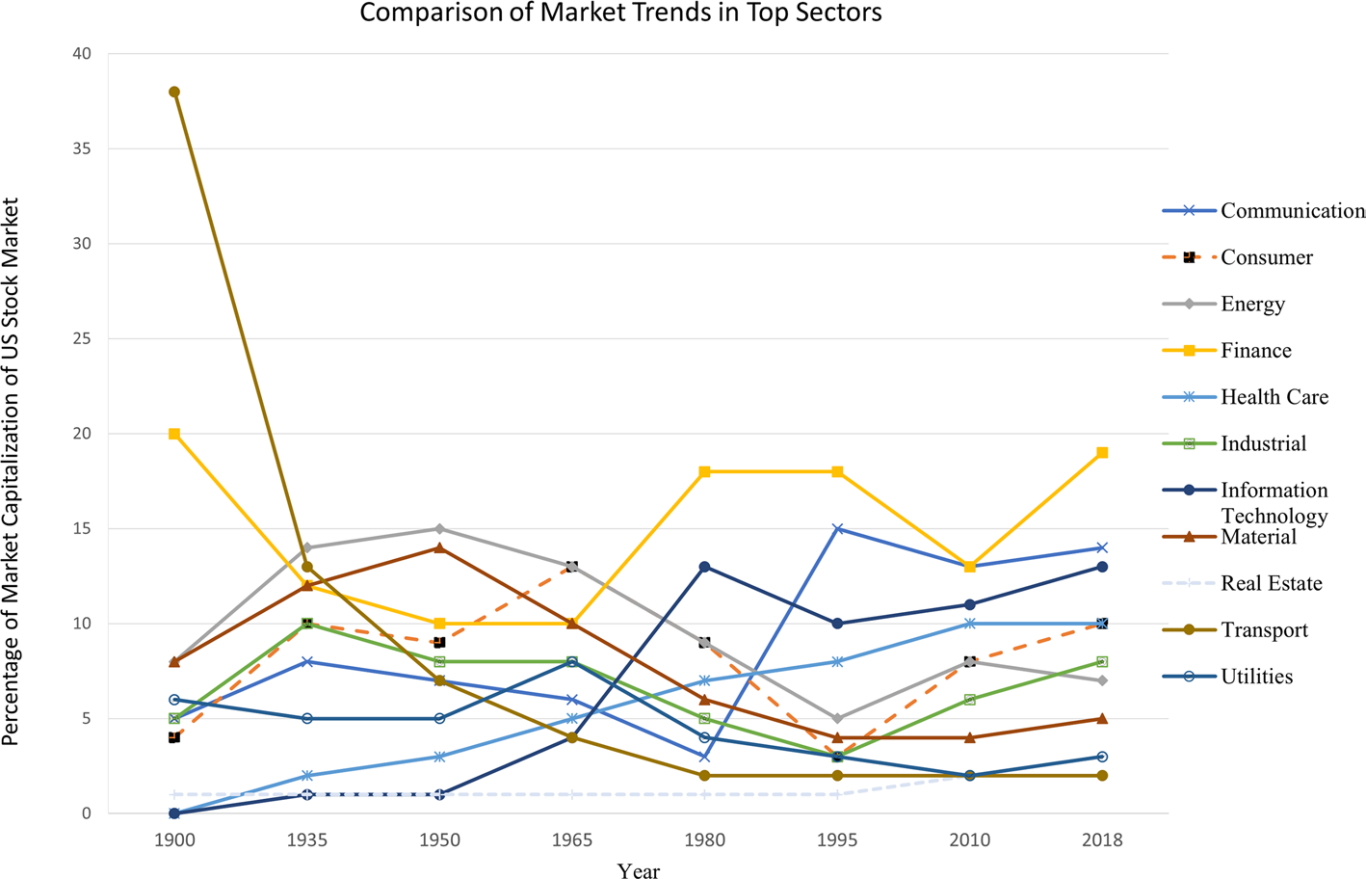
Comparison of stock market capitalization from 1900 to 2018. The *y*-axis shows the total percentage of market valuation of top sectors

Market trends constantly evolve with the changing time to fulfill customers’ needs which gives an edge to the companies in the competitive environment. For example market digitization brings new economic development by spreading the market internationally to capture a larger customer base [40]. Hence entrepreneurs are looking for constant innovations and the latest technologies being used for product development to build a successful brand and maintain a long-term relationship with the customers. Figure 7 shows the market trends of top industrial sectors based on the revenue generated in the 2022 quarter results. As shown in the figure, Technology has generated the maximum revenue followed by Retail, Finance sector, and so on [41]. In order to evaluate market trends, investors collect information about the revenue generated in these sectors, use of latest technology, measure innovation as well as evaluate the stock market trends over the years. These key features provide the investor with all the information needed to make an effective decision about investments in the business. Figure 8 highlights the percentage of market capitalization over the years for different market sectors showing the trends of market shift from 1900 to 2018 [42]. For example, the Transportation sector was significantly higher than other sections during the 1900 s but then it experienced a major decline in the 2000 s. Similarly, Information Technology had a new boom in the late 1900s and has since continued to expand. The plot shows that it is important to keep up with the market trends and other important market characteristics which make or break the business industry.

## Proposed framework

In this section, we describe the proposed framework for business success prediction including the features used for learning and the prediction framework for modeling.

**Fig. 9:**
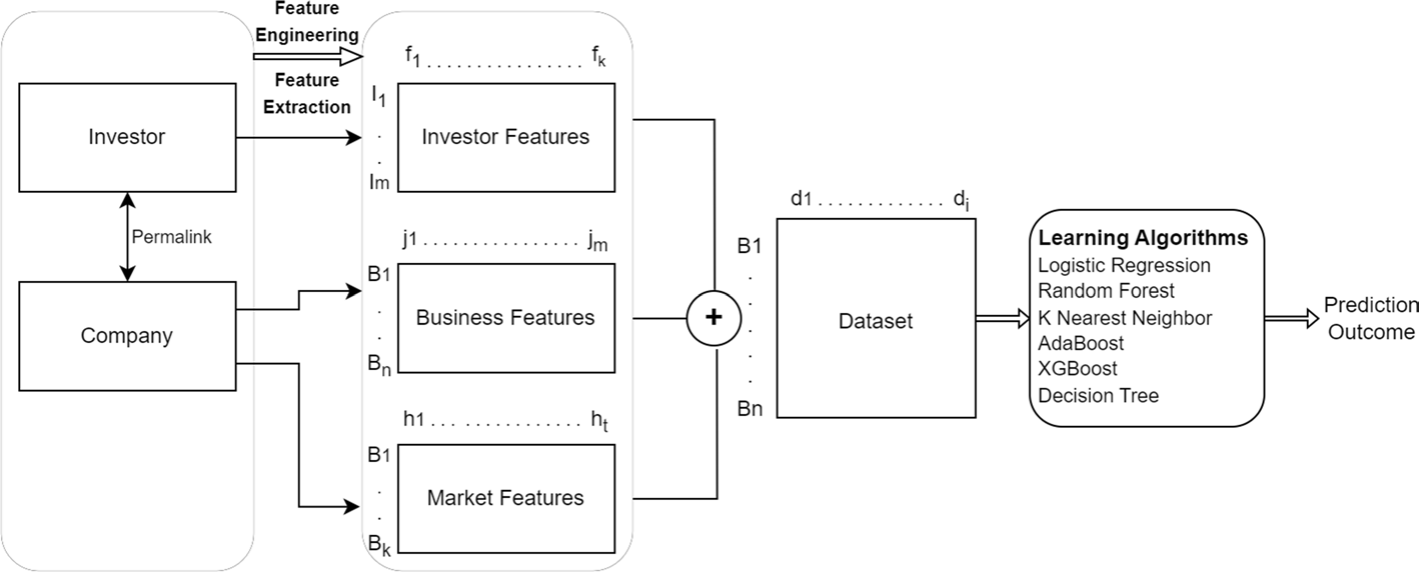
The proposed system flow chart for business success prediction. The original dataset has two tables: Company and Investor, linked by “permalink”. We first create investor features using Investor table. Business features and market features are derived from the Company table. The three features are consolidated to form IBM features to represent each business for learning and prediction

### Features for learning

The features for machine learning are the main step in the proposed framework for analyzing business success. The dataset from the company and the investor file contains detailed information about companies, Their demographic information, funding information, market sectors, and investor details. These files are exported and merged with a unique identifier known as *permalink* to extract relevant features and make them ready for modeling. Three types of features are extracted from the dataset including the investor, business, and market features which describes the correlation between IBM entities in our prediction model.

#### Investor features

In our model, we select relevant investor features including *Investor names, Funding rounds, Type of funding* and *Raised Amount*. The selection of these features is based upon how important and relevant are these features in predicting business success and their availability in our dataset. These investor features provides the domain knowledge of the financial aspects of the company and hence plays a major role in business success prediction.

Based on the available set of features, statistical analysis was performed to create new calculated set of features which provides more details on how the investments impact the company’s performance. Some available features such as the *Investor names* were one hot encoded into 14 dimensions by extracting the most common investor names (such as Angel, Venture, Bank, Technology etc. from the dataset) The investor names were extracted by counting the occurrences of each word and the top investor names were selected for feature extraction method. Other available features such as *Raised Amount* was split into 4 dimensions and scaled into *USD, millions, billions and thousands* for ease of use. Similarly, *Funding round type* and *Funding round code* provides the type of funding received by the company and the the funding code is a unique code generated for different types of funding. Using the investor names, funding information and the amount raised by investors brings in more innovations, next generation ideas and expansions into the business [4].

Based on the existing features and their critical importance to success, we calculated additional features to enhance the performance of the model and extract more information from the available dataset. Features such as *Percentage of success rate* and *Percentage of failure rate* is calculated by using the percentage of total investments made by the investor within the company. The business target (1 or 0) is used to distinguish success and failure by the investors. Similarly, we calculated the *Sum of Success Amount Raised* and *Sum of Failure Amount Raised* by the investors by adding the total amount raised and distinguishing it by the business target. Other calculated features are straightforward and includes *Number of investors*, *Number of successful companies*, *Number of failed companies*, and *Funded date* which is split into 3 dimensions. The *Total number of investors* is the count of investors who invested in each company. The final set of features including the description used in the modeling process is provided in the Table 1. The type of features are defined as either categorical or numerical and the dimension size of each feature is given after the feature extraction and encoding process.

**Table 1:** A description of Investor Features used in the prediction model

Feature names	Description	Type of feature	Dimension size afterencoding
Investor name	Name of the investor in the company	Categorical	one hot encoded- 14 dimensions
Raised amount $USD	Amount raised by investors into the company in USD	Numerical	1-D
Raised amount $m	Amount raised by investors into the company in millions	Numerical	1-D
Raised amount $b	Amount raised by investors into the company in billions	Numerical	1-D
Raised amount $k	Amount raised by investors into the company in thousands	Numerical	1-D
Number of investors	Total No. of investors in the company	Numerical	1-D
Funding round type	Type of funding received by the company (seed, angel, VC ...)	Categorical	one hot encoded - 13 dimensions
Funding round code	Funding Codes defines the code of the funding received by the company (A,B,C ...)	Categorical	one hot encoded - 6 dimensions
Percentage of success rate by company	Success rate calculated by total number of companies invested by the investor(using business target(1))	Numerical	1-D
Percentage of failure rate	Failure rate calculated by total number of companies invested by the investor(using business target(0))	Numerical	1-D
Sum of successful raised amount	Total sum of amount raised by investors based on success	Numerical	1-D
Sum of failed raised amount	Total sum of amount raised by investors based on failure	Numerical	1-D
Total raised amount	Total sum of amount raised by investors including failed and successful companies	Numerical	1-D
Average funding received	Calculated the average amount of funding received by each investor to the company	Numerical	1-D
No. of successful companies	Count of successful companies by each investor	Numerical	1-D
No. of failed companies	Count of failed companies by each investor	Numerical	1-D
Funded at year	Year at which the company received it’s funding by investor	Numerical	1-D
Funded at month	Month at which the company received it’s funding by investor	Numerical	1-D
Funded at day	Day at which the company received it’s funding by investor	Numerical	1-D

#### Business features

**Fig. 10:**
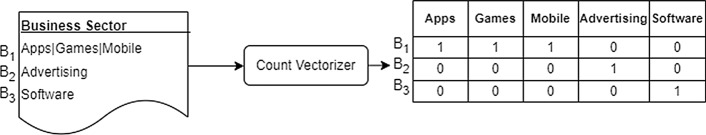
Examples of creating business sector feature using Count Vectorizer technique. Each business $B_i$ has a list of “Business Sector” tags in the dataset (left). The Count Vectorizer represents each business $B_i$ as one-hot (0/1) features, depending on whether a business has a specific “Business Sector” tag or not (right)

The business features provides detailed information about the company including the demographics, sector of business and the funding details. company’s demographic features include the *Company name, State code, Region, Country* and *Homepage URL*. The demographic information is useful in analyzing which region has the most startups and what is amount of funding received by these companies. This information provides an edge to the entrepreneurs to keep up with the sales and profit of the company [43]. Headquarter location of the firm help to attract potential customers and increase the market value. *The Business sector* contains the information about the market sector in which the company operates and is a common feature that useful in defining business information as well as market characteristics. The sector information is present in a list of categories for each company. For this reason, we use Count Vectorizer technique as a feature extraction method to tokenize and count the number of times the word occurs in the dataset after excluding the stop words from the dictionary. This technique maintains the semantic meaning of the word after transforming the sentence into tokens. Figure 10 demonstrates the example of final feature set after extraction.

Another important information included in the business feature table is the funding information about the company. This information provides details about the initial funding received by the company based on different business sectors. It has been observed that companies that receive initial funding or Venture capitalist funding usually perform better than the other companies that do not receive external funding [44]. The available funding features includes *Funding Amount, First Funding Date* and *Last Funding Date*. The *Founded at* date provides the date at which the company was founded. All these features are important to analyze the situation of the company in terms of failure and success and the date features helps to analyze the growth rate of the company. Utilizing the available list of features, we have calculated additional features to support our prediction model and highlight critical aspects of the company. With the help of the date features, we calculated *Funding duration* and scaled into days, months and years. Similarly, *Age* of the company is calculated using the *Founded at* date. The *Average duration of Funding* is calculated by finding out the average between the *first funding date* and *last funding date* by the company. Apart from this, we extracted domain information from the company features to distinguish the domain knowledge (such as.com,.net,.uk etc). The domain knowledge gained much popularity in the late 1990s when new era of internet grew across the world [45]. This led to the new rise in the businesses and many new companies were founded during the 1990s including the.com companies [46]. Hence, the *domain name* provides awareness among the users as it sets an established name within the company which helps to develop a certain amount of trust with the consumers and entrepreneurs. The final list of features for modeling is provided in the Table 2 including the description, type and dimensionality of the features after encoding. The Date features were split into month, day and year for ease of access. The following features were removed from the final dataset after analyzing the amount of incorrect information and missing values ratio within the features: *State code, region, city, company name, homepage URL and permalink*(a unique identifier, not required after merging two dataset).

**Table 2:** A description of Business Features used in the prediction model

Feature names	Description	Type of feature	Dimension size after encoding
Company domain	Domain of the company (.com,.net,.uk *etc.*)	Categorical	One hot encoded 5 dimension after encoding
Business sector	Type of business sector in which the company operates	Categorical	Count Vectorizer - 480 dimension (taken from market features)
Company status	Status of the company(closed, IPO, operating,Acquired) which later becomes the business target	Categorical	Binary label-1 dimension
Country_code	Country in which the company operates	Categorical	One hot encoded - 7 dimensions
Funding total $USD	Total amount of funding initially present in the company in USD	Numerical	1-D
Funding total $m	Total amount of funding initially present in the company in millions	Numerical	1-D
Funding total $b	Total amount of funding initially present in the company in billions	Numerical	1-D
Funding total $k	Total amount of funding initially present in the company in thousands	Numerical	1-D
Age of company	Age of company calculated using founded at date	Numerical	1-D
Average duration of funding	Average funding received by the company	Numerical	1-D
Funding duration days	The duration of the funding received by the company in days	Numerical	1-D
Funding duration months	The duration of the funding received by the company in months	Numerical	1-D
Funding duration years	The duration of the funding received by the company in years	Numerical	1-D
Founded at day	The day at which the company was founded	Numerical	1-D
Founded at month	The month at which the company was founded	Numerical	1-D
Founded at year	The year at which the company was founded	Numerical	1-D
First funding day	Day of the first funding received by the company	Numerical	1-D
First funding month	Month of the first funding received by the company	Numerical	1-D
First funding year	Year of the first funding received by the company	Numerical	1-D
Last funding day	Day of the last funding received by the company	Numerical	1-D
Last funding month	Month of the last funding received by the company	Numerical	1-D
Last funding year	Year of the last funding received by the company	Numerical	1-D
Funding round	Total rounds of funding received by the company	Numerical	Label encoded - 7 dimensions

#### Market features

Market features are an important link between the company and the investors. Entrepreneurs should consider a thorough market research in order to stay on top of the competition [47]. Accessing recent market trends in different business sectors establishes customer base within the company such that the investors can invest into the business freely and gain maximum profit from the product sales and services [48].

**Fig. 11:**
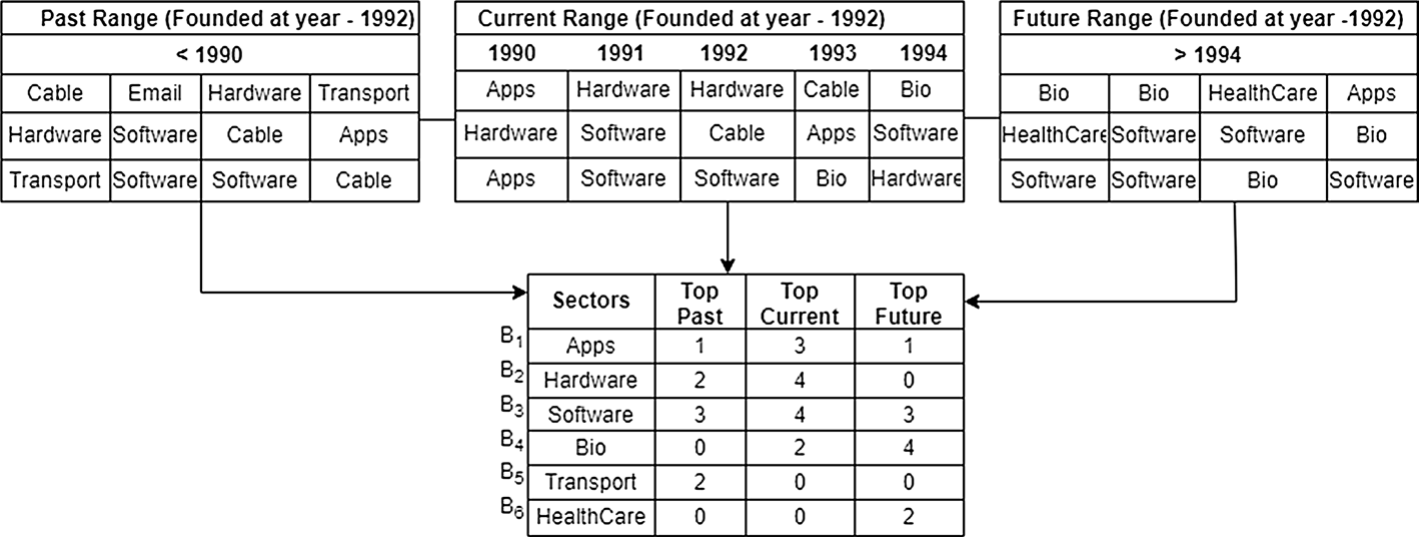
An example of creating past, current, and future ranges for market features. The top-middle table refers to top business sections at the “current” (i.e. 1990–1994). The top-left table refers to top business sections during the “past” (i.e. before 1990). The right-left table refers to top business sections in the “future” (i.e. after 1994). The table at the bottom shows number of times a sector tag appears in the companies with respect to “past”, “current”, and “future”, respectively. For example, hardware tag appeared in two sponsored companies in the past, and appeared in four sponsored companies at the current

In our dataset, market feature is available as business sectors which includes information about different markets sectors in which the company operates. The feature *Business sector* is included in the business feature table as well (as shown in Fig. 10). The *business sector* is a common feature used in the market as well as business to extract useful information about the market trends, market capabilities, funding capacity for investors and the amount received per sector within the company. In order to characterize the evolving of the market with respect to different period of time, we are creating three market features, “Top past sector”, “Top current sector”, and “Top future sector” to outline common business sectors, with respect to the founded time of each business. The motivation is to capture whether a business, when established, is falling into some hot market trends. An example of creating such features is shown in Fig. 11.

More specifically, *Top past sector*, *Top future sector* and *Top current sector* were created using *founded at date* of the company from the company feature table to distinguish past, current and future categories of the market. The calculation of *current* was based on the the year at which the company was founded and a range of two year before and two year after the founded year was considered for calculation of *current* feature. A five year range including the current founded year is used for calculating the top sectors. Figure 11 demonstrates the example of how the ranges for past, current and future are calculated from the *founded year*. Based on these ranges the final table shows the count of top sectors for each company. For the calculation of the final table, from the given range we count all the companies that have invested in the top sectors and add it to the calculated feature *Top current sector*. Similarly, the calculation of *past* includes all the years before the selected current range and the count of all the sectors in which the company invested is calculated for the feature *Top past sector*. For the feature *Top future sector*, we include all the years after the selected current range to count the top future sectors. The past, current and future are the ranges given based on the year the company was founded. These segregation of sectors provides an insight about the shifts in the market and highlights the market trends of the company with each passing year. Knowing the trends in the past, current and future sectors not only provides an advantage to the investors but also to the entrepreneurs or decision makers to keep up with the trending market and invest wisely. Another calculated feature *Funding frequency* denotes the frequency of funding received by each sector in the company. The calculation formula is given by:

1 $$\begin{aligned} \text {Funding Frequency} = \frac{\#~ of\, Sectors}{\#~ of\, Companies} \end{aligned}$$

For example how many times the company received funding for software sector. These calculated features also helps to answer the question how many companies invested in the top sectors ? Investors and entrepreneurs benefit from this information as it helps them make an effective decision about whether to move, hold or sell their investment with respect to the changes in the market.

**Table 3:** A description of Market Features used in the prediction model

Feature names	Description	Type of feature	Dimension size after encoding
Business sector	The market sector in which the business operates (for example Technology, Retail, Finance etc.)	Categorical	Tokenize using Count Vectorizer -480 dimensions
Top past sector	Calculated using count of top sectors in the past range of founded year	Numerical	1-D
Top current sector	Calculated using count of top sectors in the current range of founded year	Numerical	1-D
Top future sector	Calculated using count of top sectors in the future range of founded year	Numerical	1-D
Funding lrequency	Calculated using count of each sector divided by total number of companies	Numerical	1-D

### Prediction framework

Figure 9 briefly describes the structure of our proposed framework for business success prediction as a binary classification problem. It includes two primary database files the company and the investor which are merged together using a unique identifier known as *permalink*. In the next step, feature engineering and feature selection process is performed to extract, transform and encode investor, business and market features used in our modeling process. The textual or categorical features such as the *Business Sector* are extracted using Count Vectorizer method to preserve the semantic meaning of the words used in the feature set. Since the feature *Business Sector* includes multiple sectors in which the company operates, Count Vectorizer method counts the number of times the word occurs within the dataset. The most frequent word gets the majority count. The other categorical features are either one hot encoded or label encoded depending on the size and dimension of the features. Based on the available set of feature new features are created to support and provide additional information for modeling and predicting business success. With this available feature set, we create a final dataset which includes the concatenation of investor, business and market features tied up to each company instances as shown in the figure. This dataset is then used for modeling and predicting business success. In the next step, we apply different classification algorithms to the dataset to compare the results using the business target defined as:

2 $$\begin{aligned} y = \Biggl \{_{0, ~ \text {If company status = ``Closed''} }^{1, ~\text {If company status = ``IPO'' or ``Acquired``}} \end{aligned}$$

where *y* is the prediction value generated as the outcome of the modeling result. The business target determines the success and failure of the company.

## Experiments

In this section, we first describe the benchmark data and the experimentation settings and then report the results and comparisons of several methods on the benchmark dataset.

### Benchmark data

**Fig. 12:**
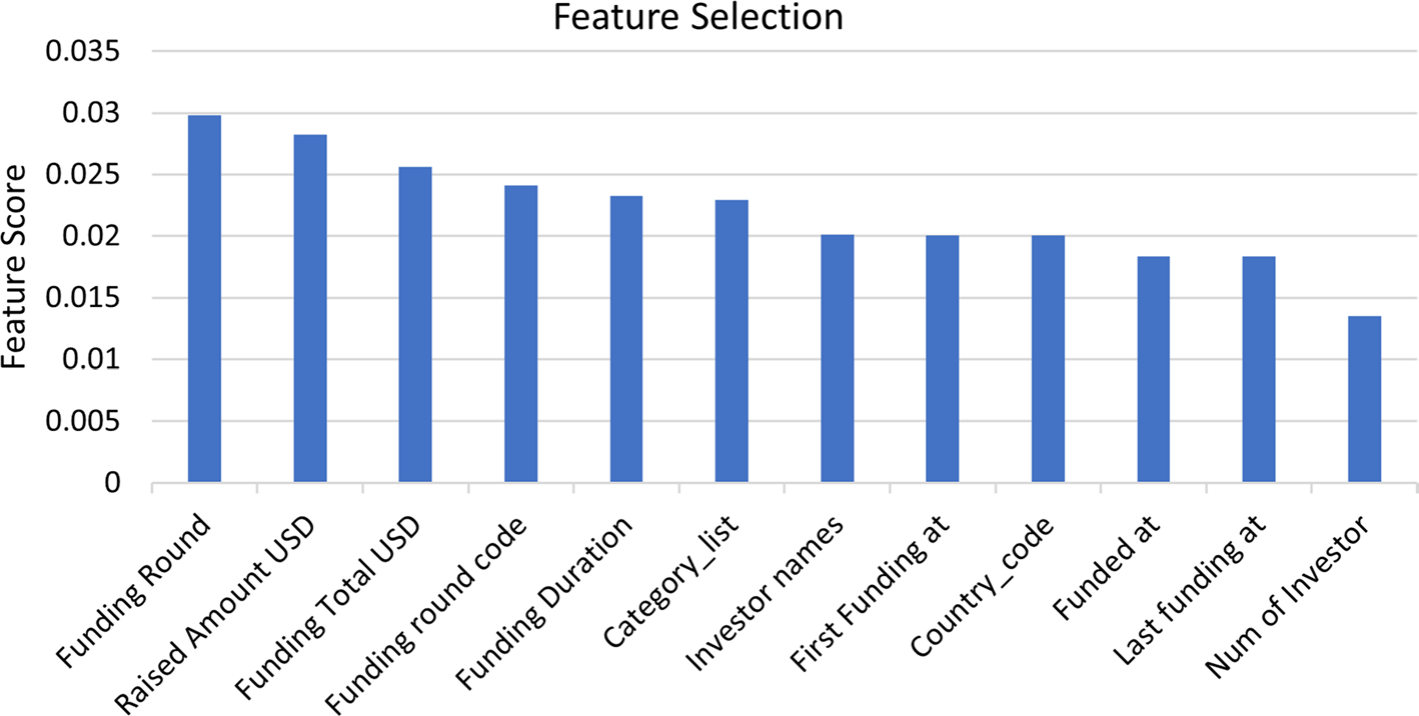
Feature selection method on benchmark dataset includes top 12 features based on the importance score to create new dependent features for modeling

The data used in the experiments were collected from a public data source .^1^ This dataset is extracted from Crunchbase.com containing 65K+ company details from 1800s to 2015. The dataset consisted of four files ”Companies”, ”Investments”, ”Acquisition” and ”Rounds”, out of which majority of the information regarding the business demographics, market sector and funding information were available in the company file and the investor information including the investor demographics, funding round code and funding round type were available in the investment file. Hence we chose company and investment file as the main data source and merged them together using a unique identifier *(Permalink)* to extract meaningful features from the two files given in the Table 4. The feature extraction includes meaningful variables from the company and the investor files. We performed a feature selection method on the selected variables using Chi-Squared and Pearson’s correlation methods to extract top categorical and numerical variables from the benchmark dataset as shown in Fig. 12. Based on the selected top 12 features, we created new dependent features with a total of 500 features in the benchmark dataset to improve the model’s performance. After feature engineering, feature extraction, and removing all companies having the status of ”operating” for accurate processing of business target, the final benchmark dataset as shown in Table 5 includes a total of 13,334 records out of which training set consists of 10,668 records and testing set consists of 2666 records divided into number of success and number of failed records with features representing each company information tied up to investor and market information as well.

**Table 4:** Simple statistics of the benchmark dataset. # “Companies” dataset lists all businesses. “Investments” dataset lists all investments investors made to the businesses

Data	# of fields	# of records
Companies	14	66,368
Investments	18	168,647

**Table 5:** Training *vs*. test split and respective class distributions (5-fold cross-validation was employed in the experiments. This table shows split of one fold)

Dataset	# of successful	# of failed	Total # of instances
Train set	5726	4942	10,668
Test set	1370	1296	2666

### Experimental settings

We implemented our experiments using the benchmark dataset provided in Table 5. A total of 13,334 instances for each company were selected after the Feature extraction, selection, and preprocessing stages. A total of 563 features related to Investor-Business-market features as shown in Tables 1, 2, 3 were used for modeling and predicting business success for our proposed method and 500 features were selected for baseline method. Eight classification learning algorithms were applied in our experiments, including Logistic Regression (LR), Decision Tree (DT), K-Nearest Neighbor (KNN), Random forest with 100 trees (RF-100), Random Forest with 200 trees (RF-200), Extreme Gradient Boosting (XGBoost), Adaptive Boosting (AdaBoost) and Sequential Neural Network (NN).

All models were built using keras and scikit-learn library in python. For training the models, we separate the dataset into two portions, training and test set using 5-fold cross-validation, where (k-1) 4-folds are used for training and 1-fold is used for testing. In the preprocessing step, textual features such as *business sector* are converted into vectors and other categorical features are converted using either one-hot encoding or label encoding process depending on the size of the feature dimension. *Business Sector* is represented as textual features due to the fact that each company can operate on multiple industry sectors instead of one. Hence we use Count Vectorizer technique for conversion.

All results are obtained via 5 repeats of 5-fold cross-validation and our experiments are carried out on the training dataset and evaluated on the testing data.

#### Baseline methods

For our baseline method, we performed all experiments using features obtained from company data file. No new features were added or selected from investor data file since many Previous studies [7, 9, 49, 50] considered only company features for predicting business success. Hence we chose company features as our baseline methods and compared the performance with our proposed IBM features to study and evaluate how different features influence the modeling results. The Table 6 demonstrates the total features selected as baseline and their dimensions after encoding. A total of 500 features after conversion were used as a baseline method for predicting business success.

For fair comparison, all the experiments were performed on the same training and testing data with same number of instances. Eight machine learning classification algorithms were used with same business target of success or failure(0 or 1) for our baseline as well as for our proposed method.

- Logistic Regression is most commonly used model for binary classification tasks and has been used in many previous researches for predicting business success [7, 51]. However logistic regression has been known to have low performance as compared to tree-based algorithms.
- Random Forest has been known to achieve higher accuracy and is robust to noisy data as shown in previous studies [52]. For our experiments, we use Random forest with 100 trees and 200 trees for comparison of results.
- Decision Tree is a straightforward classification algorithm that produces comparable results for prediction tasks [53].
- K-Nearest Neighbor works by finding similar things in close proximity to each other. Hence in dataset, we use KNN as it helps to find similar companies for entrepreneurs or investors to compare and make decisions whether to invest or not. KNN has not been explored much in the field of business prediction when compared to other machine learning models.
- XGBoost is a boosting technique that has gained tremendous popularity due to its high performance and enhanced speed in prediction tasks.
- AdaBoost [54] is another important boosting algorithms that have shown success in variety of machine learning applications such as bankruptcy prediction [55], failure prediction *etc*.
- Neural Network has been widely used for classification and regression problems due to its ability to offer better consistency and work in parallel to save processing time.

**Table 6:** Baseline Features

Feature names	Type of feature	Dimension size after encoding
Business sector	Categorical	Count Vectorizer 480- Dimensions
Company status	Categorical	Binary label (0 or 1) 1-D
Country_code	Categorical	label encoded 1-D
Average funding duration days	Numerical	1-D
Average funding duration years	Numerical	1-D
Funding rounds	Numerical	1-D
Funding total $USD,$m,$b,$k	Numerical	3-D
Funding duration days, month, year	Numerical	3-D
Founded at day, month, year	Numerical	3-D
First funding day, month, year	Numerical	3-D
Last funding day, month, year	Numerical	3-D

#### Evaluation metrics

To evaluate the quality of our prediction, we use average of area under the ROC curve as the main evaluation metric as it shows the accuracy of the binary classification model by ranking positive classes against the negative ones. In addition to this, we also employ average accuracy as another performance indicator for assessing binary classification task. We use average accuracy for estimating classifier performance since we model and forecast business success using 5 repeats of 5-fold cross-validation. Using multiple 5-fold cross-validation separates the data into 5 equal-sized blocks and repeats the process 5 times. This helps in preventing the model from any kind of bias and over-fitting.

**Fig. 13:**
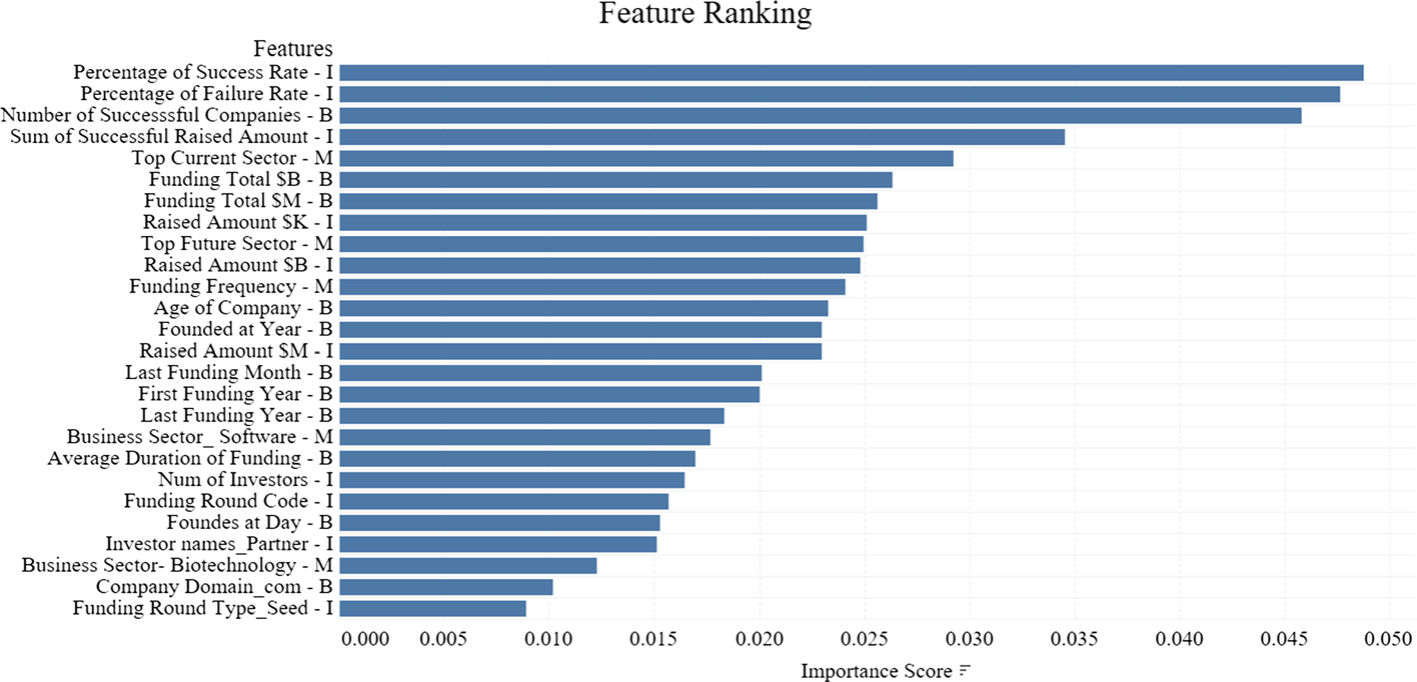
Ranking top features based on the importance score

**Fig. 14:**
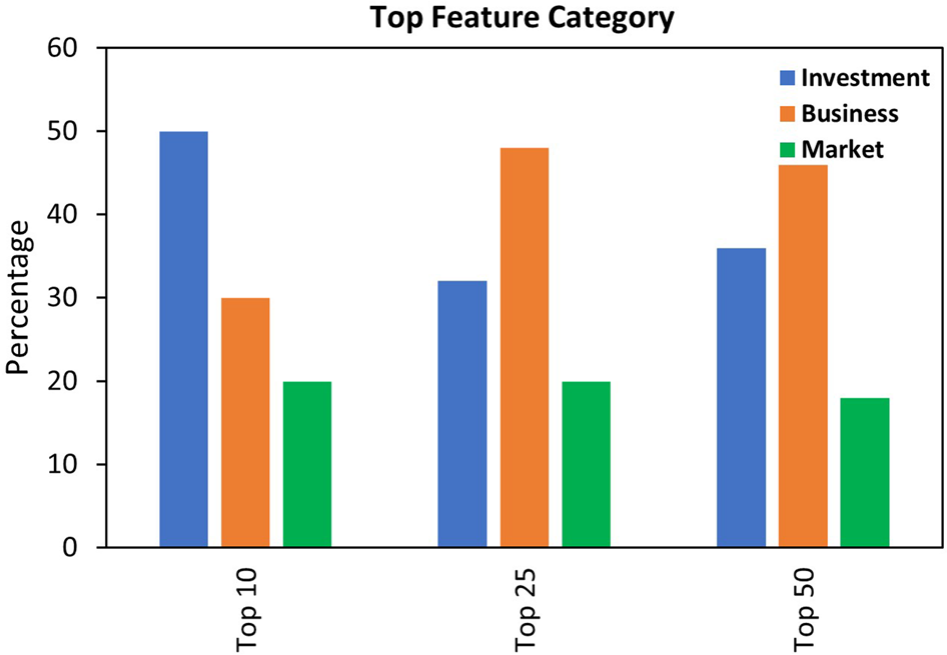
Percentage of top features in Investment, Business and Market angle

### Business success prediction results

Business prediction is a binary classification task for predicting the success of the business. Our proposed IBM interplay highlights the importance of Investment, Business and Market features for successful prediction of business. We compare our proposed method with baseline algorithms consisting of only Business features. For comparison purposes, we use the same experimental settings for baseline as well as our proposed method. The supervised learning algorithms *(LR,RF,DT,KNN,XGBoost,AdaBoost)* and neural network model use the label information to train the classifier on the train set and evaluate the results on the test set.

According to the results as shown in Table 9, we can observe that our proposed method of including IBM related features improved the performance of the models when compared to the baseline method. It has been observed that the features related to investment, business and market are important and must be considered when predicting business success. As shown in Fig. 13, the top features are ranked using the random forest classifier to train the model and get feature importance score for all the features. Each feature has been flagged with (I,B,M) representing the investment, business and market angle. The top features based on the score includes *Percentage of success rate, Number of successful companies, Top Future sector, Total raised amount, Age of the company etc.* which highlights the importance of IBM in business success prediction. Apart from this, we demonstrate the shifts in the IBM features when selecting top 10, top 25 and top 50 features demonstrating the role of each feature category during the feature ranking process. As shown in Fig. 14, Investment features cover 50% when selecting top 10 features whereas Business features remains on the top when selecting top 25 and top 50 features. Hence by considering our proposed method, the results demonstrates significant improvement when compared to the baseline.

**Fig. 15:**
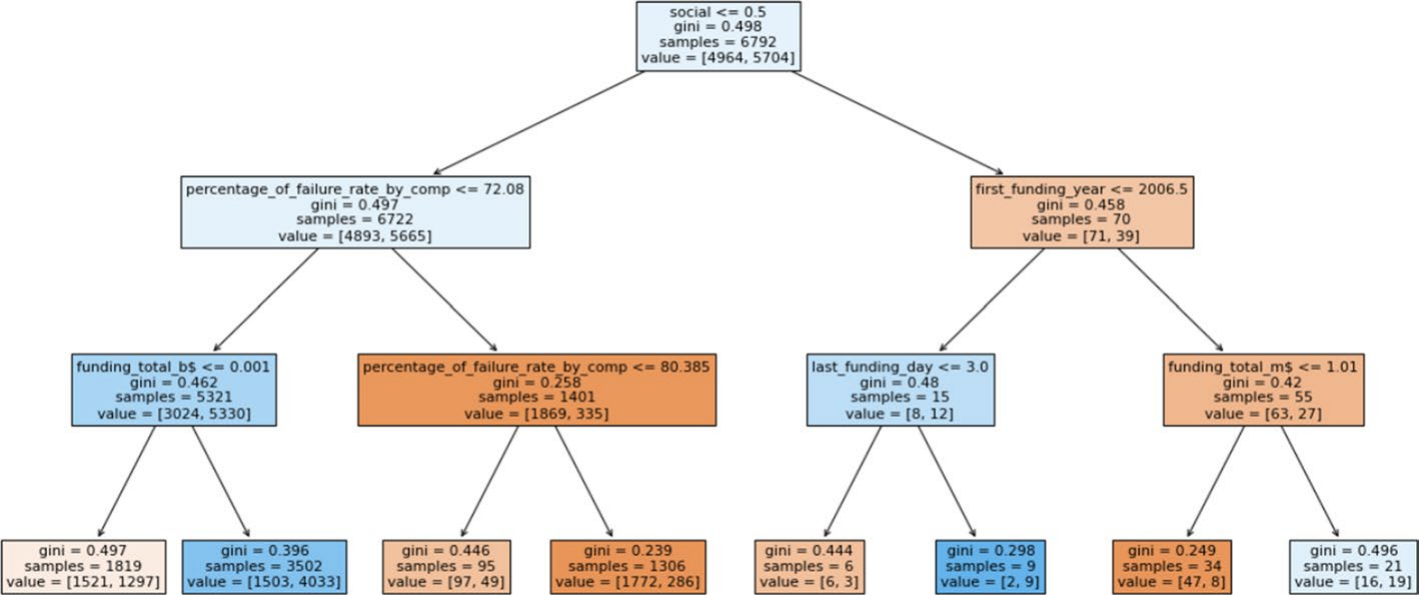
An example of a decision tree from Random Forest learned from proposed IBM features

**Fig. 16:**
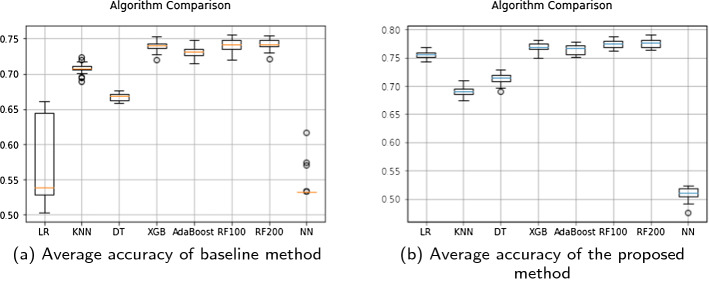
Comparisons of average accuracy of all seven learning algorithms

Among all the algorithms used for modeling, the Random Forest model and XGBoost model performed better when compared to other classification models. Random Forest with 200 trees achieved the best average accuracy of 77% and mean AUC of 85%. The second best results were obtained from XGBoost model with average accuracy of 76% and AUC of 85% followed by other models. Although accuracy of logistic regression and Neural network for baseline is low as compared to other models due to the fact that LR assumes linearity between dependant and independent variables [56]. Figure 16 shows the comparison of average accuracy for all models in baseline as well as our proposed method. Random Forest have been known to perform best in binary prediction task [57] due to it’s robust nature and efficiency in handling small and large datasets. XGBoost and AdaBoost has recently gained popularity to due it’s execution speed and high performance. Hence the results of Random forest are comparable to the boosting methods. Figure 15 demonstrates a snapshot of random forest tree with maximum depth of 3 and the first decision tree out of 200 trees estimator for simplicity of viewing.

**Fig. 17:**
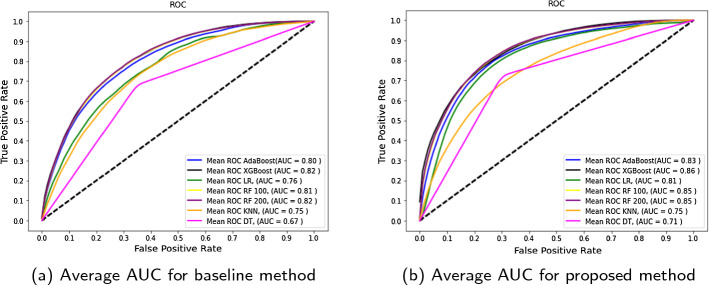
Comparison of ROC curve and AUC values for all models

In order to examine the difference between the baseline and using IBM features, Fig. 17 reports the mean ROC curves and AUC values of all models in baseline as well as our proposed model. The ROC curve is useful as it helps to understand the trade-off between the True positive and False positive ratio. We can observe an improvement of 3% in Random Forest AUC. XGBoost model has shown an improvement of 4% and overall all the algorithms have shown some improvement except for KNN. Since KNN works by identifying similar patterns, it has not been widely used in business prediction or financial analysis, hence the results may vary from other algorithms. There is a slight 0.001% difference in the accuracy of KNN algorithm baseline and proposed method. The best performance in terms of AUC is obtained by XGBoost which is followed by Random forest.

#### Type I and Type II errors

Type I and Type II errors denoted the statistical analysis of the null hypothesis in a machine learning problem. Type I error occurs when a true null hypothesis is rejected mistakenly whereas Type II error occurs when a null hypothesis is present and it fails to reject. In a binary classification problem, we denote a type I error as a false positive and Type II error as a false negative. For the prediction of business success, we classify the best-performing algorithm Random Forest-200 to evaluate statistical analysis and denote the type I and Type II error. Tables 7 and 8 demonstrate the calculated type I and type II errors for the baseline as well as the proposed IBM framework.

**Table 7:** Type I and Type II hypothesis testing error-baseline

Business success prediction (random forest-baseline)	Null hypothesis is false	Null hypothesis is true
Reject null hypothesis	863	362- False Positive (Type I error)
Accept null hypothesis	324- False Negative (Type II error)	1117

**Table 8:** Type I and Type II hypothesis testing error-proposed IBM method

Business success prediction (random forest-baseline)	Null hypothesis is false	Null hypothesis is true
Reject null hypothesis	892	359- False Positive (Type I error)
Accept null hypothesis	267- False Negative (Type II error)	1148

Our proposed IBM method outperforms all other methods in terms of accuracy and AUC scores when compared to the baseline method. Hence, we can say that considering important features from Investment, Business and Market angle and creating new features related to IBM entities are useful and necessary when predicting business success since these three entities together play an important role in analyzing successful companies and provide significant improvement in the classification performance of the models when compared to baseline method. Moreover, including the investor and market information demonstrates the capability of the company to withstand the ups and downs of the market and can be compared to a real life situation that a company faces during its life cycle of events.

**Table 9:** Business success prediction results

Method	Algorithm	Accuracy	AUC
Baseline method - 500 dimensions	Logistic regression	0.57	0.76
	Random forest-100	0.74	0.81
	Random forest-200	0.74	0.82
	Decision tree	0.66	0.67
	K-nearest neighbor	0.69	0.75
	XGBoost	0.74	0.82
	AdaBoost	0.73	0.80
	Neural network(sequential)	0.53	0.67
IBM interplay features - 563 dimensions	Logistic regression	0.75	0.81
	Random forest-100	0.77	0.85
	Random forest-200	0.77	0.85
	Decision tree	0.71	0.71
	K-nearest neighbor	0.69	0.75
	XGBoost	0.76	0.85
	AdaBoost	0.76	0.83
	Neural network(sequential)	0.52	0.60

## Conclusion and future work

In this paper, we studied the business success prediction by using an Investor-Business-Market (IBM) triangular relationship in the modeling process. We proposed to use IBM features to characterize each of the three aspects and capture their interrelation for learning and prediction. Following the proposed triangular feature relationship, we elaborated on technical details in extracting features from the benchmark dataset and created additional features based on the IBM interplay to enhance the performance of our model. Seven supervised learning algorithms are applied to the datasets by using new IBM features. Experiments and comparisons confirm that IBM feature-based methods not only outperform baseline methods in predicting business success but also provide a meaningful and transparent understanding of feature importance in the prediction. This study validates the effectiveness of computational methods, combined with carefully designed features, in the modeling and prediction of business success.

Future works could include additional features to elaborate the company and founder’s relationship with products and services offered within the company. Additional features such as investor details, founder’s information, organization, and investment relationship with products sold in the market could improve the performance of the model. However, additional features or details about the company or investor would require adding more sources from Crunchbase or TechCrunch websites that provide details about every aspect of the company, investors, founders, people, etc., to explore the advanced dataset. Apart from this, time series prediction is another exciting part of the research that can be done to analyze the growth of the company. We believe that our work demonstrates deeper insights into the available features as well as provides a capability to utilize the dataset to explore additional features using robust machine learning models.

## Data Availability

The dataset, code, and materials used in the paper will be published online (through GitHub) in the camera ready copy of the paper for public access.

## Footnotes

1 https://github.com/chenchenpan/Predict-Success-of-Startups
